# Low-Power Embedded Sensor Node for Real-Time Environmental Monitoring with On-Board Machine-Learning Inference

**DOI:** 10.3390/s26020703

**Published:** 2026-01-21

**Authors:** Manuel J. C. S. Reis

**Affiliations:** Engineering Department, Institute of Electronics and Informatics Engineering of Aveiro (IEETA), University of Trás-os-Montes e Alto Douro, 5000-801 Vila Real, Portugal; mcabral@utad.pt

**Keywords:** embedded sensor systems, environmental monitoring, edge artificial intelligence, low-power design, microcontroller architectures, LoRaWAN, on-device inference, quantised neural networks

## Abstract

This paper presents the design and optimisation of a low-power embedded sensor-node architecture for real-time environmental monitoring with on-board machine-learning inference. The proposed system integrates heterogeneous sensing elements for air quality and ambient parameters (temperature, humidity, gas concentration, and particulate matter) into a modular embedded platform based on a low-power microcontroller coupled with an energy-efficient neural inference accelerator. The design emphasises end-to-end energy optimisation through adaptive duty-cycling, hierarchical power domains, and edge-level data reduction. The embedded machine-learning layer performs lightweight event/anomaly detection via on-device multi-class classification (normal/anomalous/critical) using quantised neural models in fixed-point arithmetic. A comprehensive system-level analysis, performed via MATLAB Simulink simulations, evaluates inference accuracy, latency, and energy consumption under realistic environmental conditions. Results indicate that the proposed node achieves 94% inference accuracy, 0.87 ms latency, and an average power consumption of approximately 2.9 mWh, enabling energy-autonomous operation with hybrid solar–battery harvesting. The adaptive LoRaWAN communication strategy further reduces data transmissions by ≈88% relative to periodic reporting. The results indicate that on-device inference can reduce network traffic while maintaining reliable event detection under the evaluated operating conditions. The proposed architecture is intended to support energy-efficient environmental sensing deployments in smart-city and climate-monitoring contexts.

## 1. Introduction

Environmental monitoring plays an increasingly important role in addressing challenges such as air pollution, climate change, ecosystem degradation, and urbanisation. Traditional monitoring approaches, based on satellite or airborne remote sensing and fixed high-cost measurement stations, provide valuable data, but are often limited by sparse spatial coverage, high deployment and maintenance costs, and delays in translating measurements into actionable insights.

Recent advances in low-power embedded electronics and the Internet of Things (IoT) have enabled the deployment of distributed sensor nodes capable of capturing fine-grained environmental data at unprecedented spatial and temporal scales. Such systems are particularly relevant for long-term monitoring in urban, agricultural, and remote environments, where infrastructure availability, maintenance effort, and energy autonomy are critical constraints.

However, pervasive environmental sensing introduces significant technical challenges. Embedded sensor nodes must operate under strict energy budgets while simultaneously supporting sensing, data processing, and wireless communication. Continuously transmitting raw sensor data to centralised servers is often impractical due to radio energy costs, network bandwidth limitations, and increased latency. These constraints motivate the integration of intelligence directly at the sensing node, enabling local data processing and decision-making.

Edge artificial intelligence (edge AI) addresses this challenge by allowing machine-learning inference to be executed on resource-constrained embedded devices. In environmental monitoring applications, on-device inference allows local event or anomaly detection, adaptive sampling strategies, and selective data transmission, thereby reducing communication overhead and extending operational lifetime. Prior studies and surveys highlight the importance of quantised models, hardware–algorithm co-design, and duty-cycling techniques to achieve practical edge AI deployments on low-power platforms [1,2,3,4].

Despite this progress, many existing works address sensing, embedded processing, communication, and energy management in isolation. Fewer studies investigate these components jointly at the system level, particularly in the context of environmental monitoring, where sensing modalities, inference workload, and communication patterns are tightly coupled. As a result, the trade-offs between inference accuracy, latency, power consumption, and transmitted data volume are often insufficiently quantified within a unified design framework.

Motivated by this gap, this work presents a system-level design framework for a low-power embedded sensor node tailored to environmental monitoring with on-board machine-learning inference. The proposed architecture integrates multi-modal sensing, quantised on-device inference, adaptive duty-cycling, and event-driven wireless communication within a unified energy-aware design. Rather than proposing new sensing modalities or learning algorithms, this work focuses on the joint design and evaluation of sensing, inference, communication, and energy management within a single embedded platform. The objective is to quantify how system-level co-design affects power consumption, communication load, and event-detection latency.

The remainder of this paper is organised as follows. Section 2 reviews relevant and representative work in embedded sensor-node architectures, edge AI for IoT systems, and environmental monitoring applications. Section 3 describes the proposed system architecture and methodology, including hardware assumptions, sensing and processing pipelines, machine-learning model design, communication strategy, and energy-management approach. Section 4 presents simulation-based results that quantify trade-offs between model complexity, inference latency, power consumption, and data transmission. Section 5 discusses the main insights, limitations, and practical implications of the proposed framework, and concludes the paper.

## 2. Related Work

The domains relevant to this work span three interconnected fields: embedded sensor-node architectures for environmental monitoring, machine-learning/edge AI inference on constrained devices, and low-power communication/energy management for IoT systems. Each is reviewed in turn, with emphasis on representative trends and remaining gaps that motivate the proposed architecture.

### 2.1. Embedded Sensor-Node Architectures for Environmental Monitoring

Distributed environmental sensing has become feasible through networked sensor nodes combining multi-modal physical sensing, wireless communication, and remote data aggregation. Studies such as Laha et al. present a broad review of IoT- and sensor-based systems for air quality, water quality, and waste management monitoring, emphasising the transition from remote/centralised monitoring to embedded, low-cost, and pervasive sensor networks [5].

More recently, Narayana et al. surveyed “real-time smart monitoring of environmental…” in which sensor networks collect temperature, pressure, and other parameters, and highlight the continuing challenge of achieving both timely detection and energy efficiency in large-scale deployments [6].

Additionally, domain-specific studies such as Rathebe et al. highlight IoT-embedded sensors for radon exposure assessment as a practical example of scaling distributed sensing in resource-constrained environments [7].

These works show that while sensor node design has matured, key gaps remain in merging high-fidelity sensing with embedded data-processing (rather than raw transmission), and in optimising entire node subsystems (sensing, compute, communication, and energy) as a unified design.

### 2.2. On-Device/Edge Machine-Learning for Embedded Systems

The explosion of research in “TinyML” or on-device machine-learning has shifted attention to deploying inference tasks directly on constrained hardware rather than solely relying on centralised/cloud servers. For example, “Tiny Machine-Learning and On-Device Inference: A Survey” provides a comprehensive review of recent experimental deployments of ML models on resource-constrained devices, and characterises trade-offs around model size, quantisation, latency, and power [8].

Similarly, the “Edge AI: A Survey” frames the broader paradigm of AI at the network edge, noting the technical challenge of fulfilling strict latency, energy, and bandwidth constraints in embedded devices [3].

More recently, “A Comprehensive Survey on On-Device AI Models” articulates the data-model-system optimisation triad (data pre-processing, model compression, and system/hardware co-design) for efficient deployment of AI on devices [4].

These works provide strong evidence of the feasibility of edge inference for IoT, yet highlight that many prior works focus on the algorithmic/model side and less on full system integration (sensors ⇿ node ⇿ radio ⇿ power domain) in environmental-monitoring contexts.

### 2.3. Low-Power Communication and Energy Management for IoT Sensor Nodes

Energy efficiency—both in sensing and communication—is a critical bottleneck for embedded sensor platforms deployed in remote or off-grid scenarios. The “Survey on IoT-Edge–Cloud Continuum Systems” addresses architectural challenges, including power, communication, and distributed processing in heterogeneous networks (IoT/edge/cloud), hence relevant when designing low-power sensor nodes [9].

Likewise, “A Survey of Emerging Trends in Edge Computing” discusses the role of devices, edge nodes, and network strategies in optimising resource-use (latency, energy, and bandwidth) across large-scale deployments [9].

On the sensing side, works such as “Task Scheduling for Simultaneous IoT Sensing and Energy Harvesting: A Survey” delve into how energy-harvesting, duty-cycling, and adaptive sensing strategies support the sustainable operations of sensor nodes [10].

Despite this progress, there remains a research gap in architectures that tightly integrate embedded ML, sensor fusion, power domain management, and adaptive communication—especially tailored for environmental monitoring where conditions are variable and infrastructure may be limited.

### 2.4. Synthesis of Gaps and Motivation

From the review above, three main observations can be drawn:

1. Sensor node design remains siloed. Many works address sensing, communication, or ML separately. Few present a unified architecture that co-designs sensors, compute, communication, and energy.
2. Edge ML in environmental monitoring is under-explored. While edge ML is well studied, fewer works apply it specifically in low-power environmental-monitoring sensor nodes with real-world deployment constraints (power, connectivity, maintenance).
3. Trade-off analyses between latency, accuracy, power, and data transmission remain limited. Embedded systems researchers often evaluate one dimension (e.g., inference accuracy), but comprehensive system-level evaluations (node power consumption + latency + communication volume) are rarer.

The proposed architecture in this work addresses these gaps by presenting a modular embedded sensor node for environmental monitoring that integrates: low-power sensing, quantised on-device ML inference, adaptive duty-cycling, hierarchical power domains, and a low-energy LoRaWAN communication stack. The subsequent sections will describe the system design, simulation methods, and results illustrating trade-offs in a comprehensive manner.

## 3. System Design and Methods

### 3.1. System Overview

**Figure 1:**
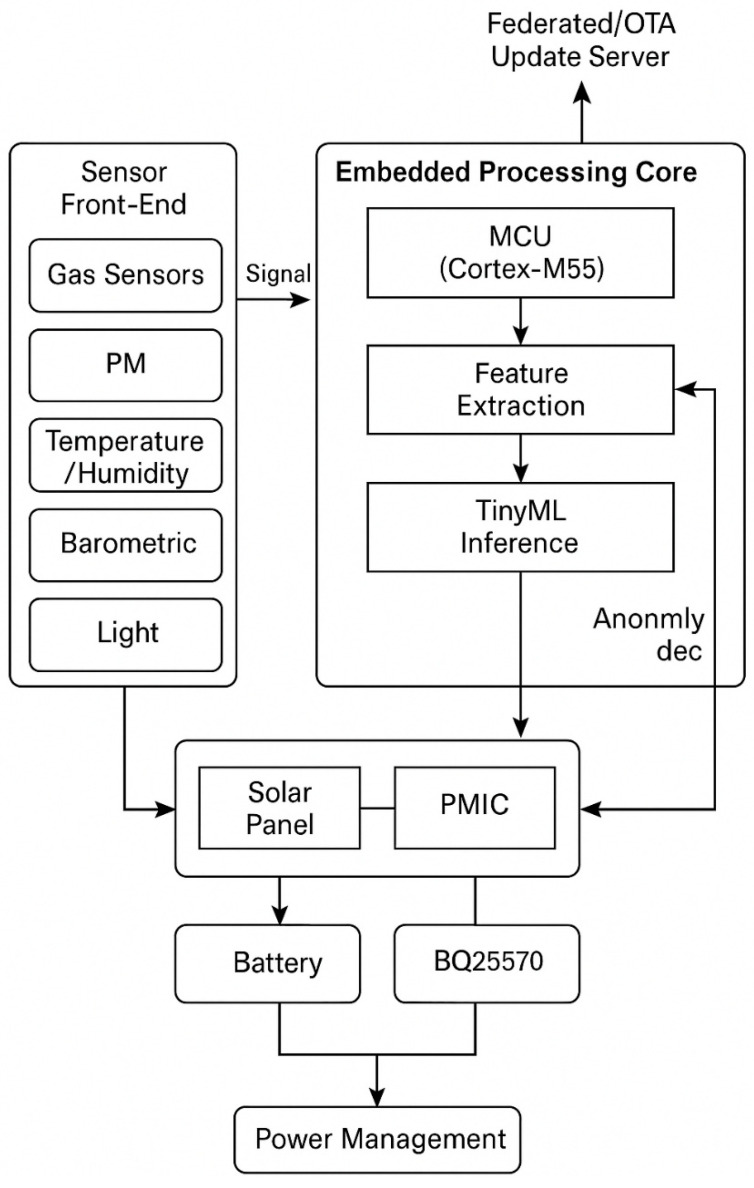
System-level architecture of the proposed low-power embedded sensor node for environmental monitoring. The platform integrates multi-modal sensing (gas, particulate, temperature, humidity, pressure, and light) with a low-power microcontroller (Cortex-M55) and a neural processing unit (Ethos-U55) for on-board machine-learning inference. Energy management is handled by a hybrid solar–battery subsystem controlled via MPPT, while adaptive duty-cycling minimises power draw. Event-triggered data are transmitted through a LoRaWAN Class A transceiver using adaptive data rate (sourced from Porto, Portugal), and maintenance or calibration can be performed locally via BLE. The modular design allows scalable deployment and future integration of federated-learning updates.

The proposed system (Figure 1) is a modular embedded platform designed for multi-parameter environmental monitoring with on-board machine-learning inference. It integrates sensing, data-processing, communication, and energy-management subsystems into a unified low-power architecture. The platform is intended for remote or semi-urban deployment scenarios where real-time or near-real-time event detection can be beneficial in several environmental monitoring scenarios.

In the proposed architecture, sensing and inference are performed locally at the node, while only summarised or event-triggered data are transmitted. This operational choice reduces radio usage and limits unnecessary data transmission compared with cloud-centric processing. This reduces network load and extends operational lifetime compared to cloud-centric architectures [3,11].

### 3.2. Hardware Platform

#### 3.2.1. Microcontroller and Processing Core

Alternative implementations could employ open-source RISC-V MCUs with integrated ML accelerators such as Kendryte K210 or Sipeed MAIX-II modules, which have been evaluated for similar TinyML workloads [12,13].

The processing core is based on an ARM Cortex-M55 microcontroller coupled with an Ethos-U55 NPU accelerator, offering native support for quantised (INT8) inference with ultra-low power draw (low-mW-level active inference power, device-class dependent). This configuration provides a balance between computational throughput and energy efficiency, ideal for environmental edge applications [8,14,15].

To anchor the compute subsystem to a realisable target, we consider a Cortex-M55 + Ethos-U55 class device (e.g., from the Ensemble E7 family) operating with a representative CPU/NPU clock of approximately 400 MHz and a nominal supply voltage of about 1.1 V for INT8 inference. Under such operating conditions, active inference power for the compute domain is typically in the tens-of-milliwatts range, while inference latencies remain in the sub-millisecond regime, yielding an energy per inference on the order of a few microjoules, consistent with the values used to calibrate the system-level simulations in this study.

#### 3.2.2. Sensing Subsystem

The sensing front-end includes the following:

- Gas sensors (CO, NO_2_, O_3_) via electrochemical elements;
- Particulate matter sensors (PM_2.5_/PM_10_) using laser-scattering;
- Temperature and humidity sensors (SHT45 series);
- Barometric pressure (BMP390) and ambient light sensors.

All sensors are interfaced through I^2^C and SPI buses with time-multiplexed sampling to minimise bus contention and enable selective power gating. Calibration coefficients are stored locally in flash memory, and signal conditioning employs low-noise amplifiers and digital filters (Butterworth 2nd-order) implemented in firmware.

It should be noted that particulate matter sensors based on laser-scattering typically require a stabilisation and sufficient sampling interval to produce representative measurements. Therefore, the short sensing burst duration (≈2 s) adopted in the simulations should be interpreted as an optimistic lower bound on sensing energy, rather than as a finalised operational configuration for PM sensing.

The sensor combination is inspired by well-documented, energy-aware air quality platforms, including the OpenSense Zurich mobile sensing project and datasets, recent reviews of long-term low-cost AQ networks, and modern open-source nodes such as AQuality32 [16,17,18,19].

Particulate sensors based on laser-scattering may require warm-up and sufficient sampling time to ensure representative readings. The current simulation assumes an idealised duty-cycling regime; in practice, PM sensing would incorporate a stabilisation interval (or a lower-frequency continuous mode), which will increase the sensing energy budget. This will be explicitly validated and parameterized in the planned hardware prototype.

### 3.3. Embedded Processing and ML Inference

#### 3.3.1. Signal Pre-Processing

Raw sensor data undergo a low-pass filtering stage followed by z-score normalisation and feature extraction (mean, variance, skewness, and kurtosis) computed over sliding windows (30 s). The feature vectors are then fed to the inference engine. All pre-processing runs on the MCU without accelerator involvement to minimise wake-up overhead.

#### 3.3.2. Machine-Learning Model

The on-board inference employs a quantised neural-network (QNN) classifier for event/anomaly detection, producing three operational states (normal/anomalous/critical). The model architecture comprises the following:

- Input layer (8–12 features);
- Two dense layers (32 → 16 neurons, ReLU);
- Output softmax (three classes: normal/anomalous/critical).

Weights are stored in on-chip SRAM and executed in fixed-point INT8. Model size ≈ 12 kB.

This model design aligns with proven low-power TinyML strategies [4,8].

Model updates are managed via federated-learning compatibility, enabling future OTA (over-the-air) updates when connectivity allows a relevant step towards supporting adaptive long-term deployments.

Moment-based descriptors (mean/variance/skewness/kurtosis) provide a compact representation of baseline level, variability, and transient asymmetry/heavy tails, which are typical signatures of short pollution events and sensor perturbations. This choice was driven by computational simplicity and robustness under INT8 inference.

### 3.4. Communication Subsystem

For wide-area, low-data-rate communication, the node employs LoRaWAN Class A operating at 868 MHz (EU868). A sub-GHz transceiver (Semtech SX1276; sourced from Geneva, Switzerland) supports adaptive data rate (ADR) to dynamically balance link reliability and energy. The stack is implemented using the open-source LoRaMAC-node firmware, with duty-cycle enforcement per ETSI 300.220.

Transmission scheduling follows an event-driven policy: packets are only transmitted when the embedded inference detects anomalies beyond adaptive thresholds, thereby reducing network traffic up to 90% compared with periodic reporting [19].

Local short-range communication (BLE 5.2) is also integrated for maintenance or calibration tasks.

### 3.5. Energy Management

Energy autonomy is achieved through a hybrid energy-harvesting subsystem combining a small photovoltaic panel (≈1 Wpeak) and a 1200 mAh Li-ion battery. The power-management IC (BQ25570) controls maximum-power-point tracking and system-load regulation.

Energy optimisation relies on dynamic power domains and adaptive duty-cycling:

- The MCU and sensors operate in sleep mode for >95% of the time;
- ML inference and LoRa transmissions are triggered asynchronously;
- The radio and NPU domains are powered only when required.

These strategies are consistent with best practices identified in energy-aware IoT reviews [9,10].

### 3.6. Simulation Framework

This work focuses on a system-level design and simulation-based evaluation of a low-power embedded sensor node architecture. While no physical hardware prototype is implemented at this stage, the simulation framework allows early-stage exploration of energy–latency–communication trade-offs that would be costly and time-consuming to assess exclusively through iterative prototyping.

Given that no physical prototype is yet implemented, a detailed simulation framework has been developed in MATLAB R2023b (MathWorks, Natick, MA, USA), Simulink R2023b + Simscape Electrical R2023b to estimate subsystem power profiles, communication latency, and inference timing:

- Sensor sampling and ADC models reflect datasheet-specified current draws;
- MCU/NPU activity is modelled using cycle-accurate estimates from ARM CMSIS Power Estimator;
- LoRaWAN stack simulation incorporates stochastic channel models for packet loss and ADR adaptation;
- Battery and solar input are simulated using variable irradiance profiles (data from MeteoBlue for the UTAD region).

MATLAB Simulink was selected as the primary modelling environment due to its native integration with Simscape Electrical and ARM CMSIS libraries, which allow cycle-accurate estimation of MCU/NPU activity and detailed component-level energy profiling. Compared with open-source alternatives such as OpenModelica or ns-3, Simulink provides a unified, time-synchronised co-simulation framework for analogue, digital, and communication subsystems. This integration facilitates end-to-end energy–latency trade-off analysis within a single environment, ensuring numerical consistency across power, computation, and networking models.

MCU/NPU activity is estimated using ARM tooling (CMSIS-based power estimation) to obtain reference cycle and power figures for the target class of Cortex-M55 + Ethos-U55 systems. For the parametric sweeps (model size vs. latency/energy), inference cost is then scaled from a calibrated reference profile, preserving consistency with the cycle/energy baseline rather than assuming an arbitrary fixed cost.

Simulation results provide a basis for evaluating trade-offs between sampling interval, model complexity, inference accuracy, and power consumption. These results are presented in Section 4.

### 3.7. Design Summary

Table 1 summarises key design parameters and their relevance to system-level optimisation.

## 4. Simulation Results and Discussion

### 4.1. Simulation Setup

System-level simulations were carried out in MATLAB Simulink R2024b with Simscape Electrical extensions to model the electrical power domain, LoRaWAN transmission stack, and duty-cycle scheduler. The embedded processor (ARM Cortex-M55 + Ethos-U55) was represented using the CMSIS Power Estimator library, providing current draw per instruction type. Sensor models were parameterised according to vendor datasheets (Alphasense CO B4, PMS7003, SHT45, BMP390).

Environmental stimuli were simulated using hourly air quality data (temperature, humidity, PM_2.5_, CO, and NO_2_) from the Copernicus Atmosphere Monitoring Service dataset for the UTAD region.

The TinyML model was trained offline on a subset of the UCI Air Quality Dataset and quantised to INT8 precision using TensorFlow-Lite Micro. The resulting model (≈12 kB) was deployed within a Simulink subsystem, and inference latency was modelled as a fixed computation cost proportional to network depth and feature-vector size.

Hourly CAMS time series are used to generate a realistic baseline environmental trajectory for the UTAD region. To match the 30 s feature window used by the embedded pipeline, the hourly trajectories are converted into 30 s samples via interpolation and a lightweight stochastic micro-variability model (to represent intra-hour fluctuations and sensor noise). Features (mean/variance/skewness/kurtosis) are computed on these reconstructed 30 s windows. The classifier itself is trained offline on a curated subset of the UCI Air Quality dataset; CAMS-driven stimuli are used for system-level evaluation (power/latency/communication triggering) rather than as a direct supervised training source.

### 4.2. Power-Consumption Profile

**Figure 2:**
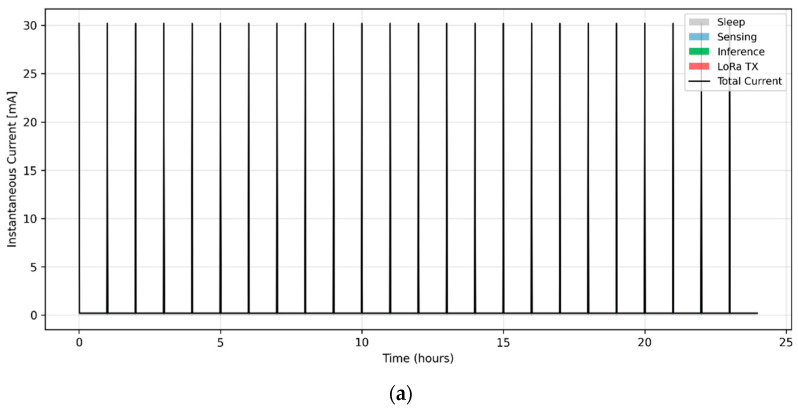

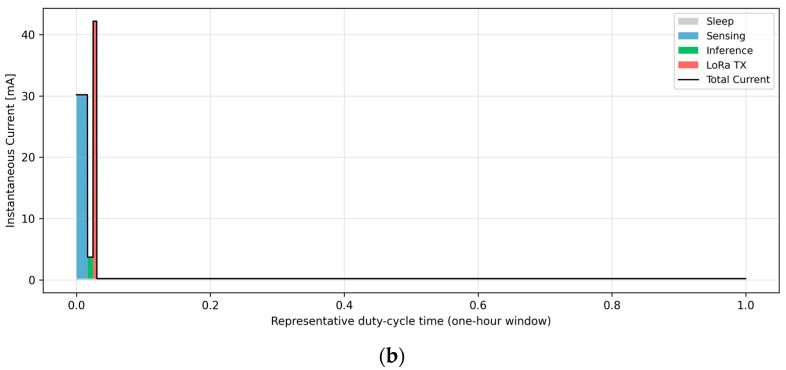
Simulated power-consumption profile of the embedded sensor node under duty-cycled operation. (**a**) One-hour representative operating cycle, where short-duration current peaks correspond to sensing, inference, and LoRaWAN transmission events superimposed on a low-power sleep baseline. (**b**) Zoomed view of the same one-hour cycle highlighting the temporal separation between sensing, inference, and communication phases. The narrow spikes represent short active intervals rather than grid lines; for visualisation purposes, some events are visually expanded while energy calculations are performed using the true durations.

Figure 2 illustrates the simulated power profile of the proposed sensor node during a continuous 24-h operating cycle. The node remains in deep-sleep mode for most of the time, drawing less than 200 µA with only the real-time clock active. During each sampling period, the system wakes for approximately 2 s to perform sensing, consuming around 30 mA as the gas and particulate sensors are powered and data are acquired. Immediately after each acquisition, the on-board inference module is activated for roughly 50 ms, drawing an additional 3.5 mA to execute the quantised neural-network model. Infrequent LoRaWAN transmission events, triggered only when anomaly thresholds are exceeded, consume approximately 42 mA for 0.8 s per transmission.

Integrating these activity phases yields a mean hourly energy consumption of 2.9 mWh, which corresponds to a projected autonomy of about 17 days when powered solely by a 1200 mAh Li-ion battery. Under nominal solar irradiance, the hybrid energy-harvesting subsystem (1 W photovoltaic panel providing ≈ 200 mWh day^−1^) can sustain indefinite operation, confirming the feasibility of energy-autonomous deployment for long-term environmental monitoring.

Comparable low-power nodes in the literature [3,20] report similar per-cycle power envelopes, confirming the feasibility of the proposed configuration.

To quantify the energy distribution among subsystems, Table 2 summarises the mean power and corresponding share of total consumption derived from the 24-h simulation.

### 4.3. Inference Accuracy and Latency

The quantised model achieved an accuracy of 94.2% and an F1-score of 0.91 on the simulated anomaly detection task, compared with 96.7% for the baseline floating-point model. The latency per inference was 0.87 ms, within the real-time window (<1 s) required for responsive detection.

Performance degradation due to quantisation (<3%) is consistent with observations from TinyML studies [4,8]. The energy cost per inference was estimated at 6.1 µJ, equivalent to <0.2% of the total hourly energy budget. Inference energy is computed as $E_{\inf}=P_{\inf}\;\times \;t_{\inf}$, where $t_{\inf}$ is the simulated inference time and $P_{\inf}$ is the active power of the compute domain during inference (MCU+NPU). Using $t_{\inf}$ = 0.87 ms and $P_{\inf}$ = 7.0 mW (baseline estimate for the compute domain under INT8 inference), we obtain $E_{\inf}$ ≈ 6.1 μJ. The baseline $P_{\inf}$ is derived from the CMSIS-based estimation workflow for a Cortex-M55 + Ethos-U55 class system.

### 4.4. Communication Efficiency

Adopting an event-driven LoRaWAN strategy significantly reduced network traffic. Only 5% of time-steps triggered transmissions (i.e., anomaly or threshold event), resulting in an 88% reduction in packet count compared to periodic (1-min) reporting.

Transmission reliability was evaluated under variable channel models (path-loss exponent 2.7–3.2, 10 dB fading margin). Packet delivery ratio (PDR) remained above 97% for distances up to 2 km LoS. These results are consistent with the objectives of LoRaWAN Adaptive Data Rate (ADR) schemes in IoT environmental sensing deployments, where ADR dynamically adjusts data rate, airtime, and energy consumption to improve battery lifetime and network efficiency, as reported in the literature [21].

### 4.5. Design Trade-Off Analysis

A parametric sweep of inference frequency (0.1 Hz–1 Hz) and model size (8 kB–20 kB) reveals the following trends:

- Increasing model size improves accuracy (+1.8%) but increases latency (+45%) and energy (+60%).
- Sampling every 60 s balances detection resolution and battery life (approximately 17 days autonomy).
- Hybrid solar input can offset higher sampling without compromising autonomy.

Such trade-offs illustrate the practical design flexibility achievable through hardware–software co-optimisation in embedded-AI systems [11].

**Figure 3:**
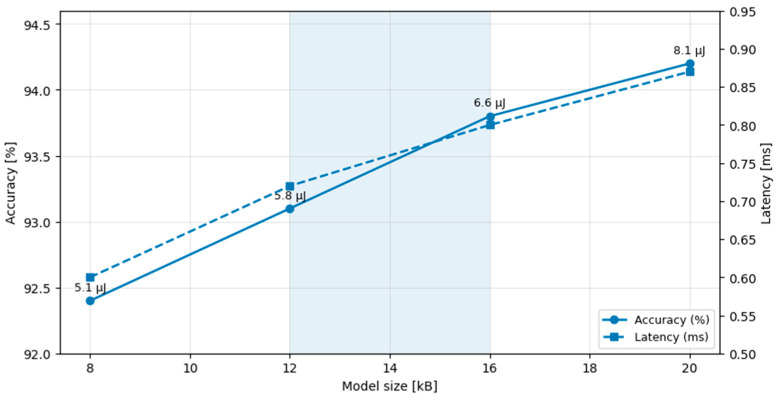
Simulated trade-off between quantised model size and inference performance metrics. Increasing the model size from 8 kB to 20 kB improves classification accuracy by approximately 1.8%, while increasing inference latency by approximately 45% and energy consumption by approximately 60%. The optimal operational region (highlighted) balances inference precision and energy autonomy for low-power embedded deployment. The shaded blue region highlights the selected operating range of model sizes, representing a favourable trade-off between accuracy improvement and inference latency.

Figure 3 illustrates the simulated trade-offs between model complexity, inference accuracy, latency, and energy consumption, highlighting the Pareto-type relationships that govern on-device neural inference. The figure presents a representative operating envelope in which increasing model size improves accuracy at the expense of higher latency and energy cost. Additional parameters, including sampling rate and decision thresholds, were also explored during simulation but are omitted for clarity, as they exhibit similar Pareto-type trends.

### 4.6. Discussion

The simulation results indicate that on-device inference can be executed within a few milliwatts of average power under the considered operating assumptions. The architecture meets edge AI design targets of sub-1 ms latency, >90% accuracy, and >10× data-traffic reduction.

Compared with conventional periodic-reporting sensor nodes, the proposed architecture prioritises reduced communication frequency and local decision-making, resulting in lower average power consumption and decreased network load. These differences are quantified in Section 4 through system-level simulations rather than direct hardware benchmarking.

Future implementations will focus on hardware validation and the integration of federated-learning mechanisms to enable adaptive model updates at the edge without full retraining. Such approaches follow recent frameworks on secure and adaptive federated learning for IoT environments and 6G-enabled sensor networks, which emphasise privacy preservation, communication efficiency, and continual adaptation in distributed settings [22,23,24].

Although direct hardware benchmarking is beyond the scope of this simulation-based study, Table 3 provides a quantitative comparison with a conventional periodic-reporting sensor-node architecture to contextualise the potential benefits of embedded intelligence. The comparison focuses on key performance metrics, including power consumption, communication load, and operational autonomy.

## 5. Conclusions and Future Work

This paper presented a system-level design and simulation-based evaluation of a low-power embedded sensor node for environmental monitoring with on-board machine-learning inference. The proposed architecture integrates heterogeneous sensing, local intelligence, and adaptive wireless communication within a unified, energy-aware framework. Simulation results indicate that the node can achieve sub-millisecond inference latency, detection accuracy above 90%, and an approximate 88% reduction in network transmissions compared with conventional periodic-reporting schemes.

The study shows the feasibility of integrating sensing, embedded inference, and communication under strict energy constraints through three complementary design aspects: (i) hardware–software co-optimisation using quantised neural inference on Cortex-M55 + Ethos-U55 class platforms to reduce computational energy; (ii) adaptive duty-cycling and hierarchical power domains to support long-term autonomy under hybrid solar–battery operation; (iii) event-driven LoRaWAN communication, in which anomaly-triggered transmissions substantially lower power consumption and spectrum usage. Together, these elements illustrate how embedded intelligence can be practically deployed to enable in situ analytics in resource-constrained IoT environments.

Beyond the presented results, the proposed methodology and parameterisation provide a reproducible framework for evaluating trade-offs between inference accuracy, latency, power consumption, and communication load in embedded-AI sensor nodes. While the present work focuses on simulation-based analysis, the framework is readily extensible to hardware validation and comparative studies across different sensing and deployment scenarios.

In practical terms, the architecture can be adapted to a range of environmental and urban-monitoring applications, including vineyard and orchard microclimate monitoring, detection of air pollution hotspots in dense urban areas, and autonomous supervision of indoor air quality or industrial emissions. These use cases share common requirements for long-term, low-maintenance operation, local intelligence, and minimal network bandwidth usage, which are directly addressed by the proposed design.

Several limitations are acknowledged, including electrochemical sensor cross-sensitivity (e.g., humidity and interfering gases), long-term drift, and warm-up or stabilisation effects. These factors may influence feature stability and decision thresholds and will be explicitly addressed through calibration strategies and experimental validation in future prototype implementations.

Future work will extend this simulation-based study into a hardware prototype, including board-level integration, firmware implementation, and outdoor field testing under variable meteorological conditions to experimentally validate the simulated power profiles, sensing constraints, and communication performance. Further research will also investigate secure and adaptive federated-learning frameworks to support continual on-device model updates without centralised retraining, in line with recent advances in privacy-preserving edge learning for IoT systems [22,23]. Additional directions include the integration of neuromorphic sensing modalities, ultra-low-power analog inference techniques, and cross-domain energy-harvesting optimisation to support scalable deployments in smart-city and precision-agriculture scenarios. In particular, the stabilisation and sampling requirements of laser-scattering particulate matter sensors will be explicitly characterised during future hardware prototyping, as they directly impact the achievable duty-cycling and sensing energy budget.

Finally, this work aligns with broader sustainability objectives, including those articulated in the European Green Deal and the United Nations Sustainable Development Goals (SDGs 11 and 13). By reducing average power consumption, communication frequency, and maintenance requirements, energy-autonomous embedded sensor nodes can contribute to lower operational energy demand and lifecycle emissions, supporting sustainable and resilient environmental sensing infrastructures.

## Figures and Tables

**Table 1:** Summary of the main hardware and subsystem design parameters of the proposed low-power embedded sensor node for environmental monitoring. Each component was selected to balance sensing fidelity, computational capability, and energy efficiency, ensuring compatibility with duty-cycled operation and edge-inference requirements.

Subsystem	Key Component	Typical Power	Design Rationale
MCU/NPU	Cortex-M55 + Ethos-U55	<2 mW active	Energy-efficient INT8 inference
Gas sensors	EC series (Alphasense)	≈30 mW	Selective activation via gating
PM sensor	PMS7003	≤100 mW @ 1 Hz	Duty-cycled operation
Communication	LoRaWAN SX1276	≈50 mW TX	ADR and event-driven
Energy	BQ25570 + Li-ion 1200 mAh	–	Hybrid solar + battery autonomy

**Table 2:** Energy consumption breakdown of the proposed sensor node (24 h simulation). The data quantify the relative contributions of sensing, inference, communication, and idle phases to the overall power budget.

Subsystem	Mean Power (mW)	Energy Share (%)
Sensors (gas, PM, etc.)	1.95	67.2
Inference (MCU + NPU)	0.20	6.9
Communication (LoRa TX)	0.60	20.7
System idle and housekeeping	0.15	5.2
Total	2.90 mW h^−1^	100%

**Table 3:** Quantitative comparison between a conventional periodic-reporting environmental sensor node and the proposed low-power embedded-intelligence node. The latter achieves a roughly 66% reduction in mean power consumption, 88% fewer transmissions, and triple battery-only autonomy, while maintaining 94% anomaly-detection accuracy through on-board inference.

Metric	Conventional Periodic Node	Proposed Embedded-Intelligence Node	Improvement
Mean power consumption [mWh]	8.5	2.9	≈66% reduction
Average inference latency [ms]	– (none, cloud-processed)	0.87	Real-time local processing
Transmission frequency	1 packet min^−1^ (fixed)	Event-driven (~5% of cycles)	≈88% fewer transmissions
Detection accuracy [%]	≈91 (cloud model)	≈94 (on-device quantised model)	+3 percentage points
Data volume sent per day [kB]	1440 (≈1 pkt min^−1^)	≈170	≈8× reduction
Operational autonomy (1200 mAh cell)	≈6 days	≈17 days (or continuous with solar)	≈3× extension

## Data Availability

The data supporting this study’s findings are available upon reasonable request from the corresponding author. Sharing the data via direct communication ensures adequate support for replication or verification efforts and allows for appropriate guidance in its use and interpretation.

## References

[1] Aral A. (2024). The Promise of Neuromorphic Edge AI for Rural Environmental Monitoring. Environ. Data Sci..

[2] Olawade D.B., Wada O.Z., Ige A.O., Egbewole B.I., Olojo A., Oladapo B.I. (2024). Artificial Intelligence in Environmental Monitoring: Advancements, Challenges, and Future Directions. Hyg. Environ. Health Adv..

[3] Singh R., Gill S.S. (2023). Edge AI: A Survey. Internet Things Cyber-Phys. Syst..

[4] Wang X., Tang Z., Guo J., Meng T., Wang C., Wang T., Jia W. (2025). Empowering Edge Intelligence: A Comprehensive Survey on On-Device AI Models. ACM Comput. Surv..

[5] Laha S.R., Pattanayak B.K., Pattnaik S. (2022). Advancement of Environmental Monitoring System Using IoT and Sensor: A Comprehensive Analysis. AIMS Environ. Sci..

[6] Narayana T.L., Venkatesh C., Kiran A., J C.B., Kumar A., Khan S.B., Almusharraf A., Quasim M.T. (2024). Advances in Real Time Smart Monitoring of Environmental Parameters Using IoT and Sensors. Heliyon.

[7] Rathebe P.C., Kholopo M. (2025). Radon Exposure Assessment: IoT-Embedded Sensors. Sensors.

[8] Heydari S., Mahmoud Q.H. (2025). Tiny Machine Learning and On-Device Inference: A Survey of Applications, Challenges, and Future Directions. Sensors.

[9] Gkonis P., Giannopoulos A., Trakadas P., Masip-Bruin X., D’Andria F. (2023). A Survey on IoT-Edge-Cloud Continuum Systems: Status, Challenges, Use Cases, and Open Issues. Future Internet.

[10] Sandhu M.M., Khalifa S., Jurdak R., Portmann M. (2020). Task Scheduling for Simultaneous IoT Sensing and Energy Harvesting: A Survey and Critical Analysis. arXiv.

[11] Saha S., Banerjee K., Ghosh S., Mitra S., Pal D. (2023). AI-Driven Edge Computing for IoT: A Comprehensive Survey and Future Directions. Int. J. Adv. Res. Sci. Commun. Technol..

[12] Tyagi A., Jeyapaul R., Zhou C., Whatmough P., Zhu Y. (2024). Characterizing Soft-Error Resiliency in Arm’s Ethos-U55 Embedded Machine Learning Accelerator. Proceedings of the 2024 IEEE International Symposium on Performance Analysis of Systems and Software (ISPASS).

[13] Moss A., Lee H., Xun L., Min C., Kawsar F., Montanari A. (2023). Ultra-Low Power DNN Accelerators for IoT: Resource Characterization of the MAX78000. Proceedings of the 20th ACM Conference on Embedded Networked Sensor Systems, New York, NY, USA, 24 January 2023.

[14] Faheem M. (2025). Energy Efficient Neural Architectures for TinyML Applications. Int. J. Sci. Res. Mod. Technol..

[15] Chaoraingern J., Numsomran A. (2025). Embedded Sensor Data Fusion and TinyML for Real-Time Remaining Useful Life Estimation of UAV Li Polymer Batteries. Sensors.

[16] Li J.J., Faltings B., Saukh O., Hasenfratz D., Beutel J. Sensing the Air We Breathe—The OpenSense Zurich Dataset. Proceedings of the AAAI Conference on Artificial Intelligence.

[17] Maag B., Hasenfratz D., Saukh O., Zhou Z., Walser C., Beutel J., Thiele L. Ozone and Carbon Monoxide Dataset Collected by the OpenSense Zurich Mobile Sensor Network. ETH Zürich Research Collection, Zurich, Switzerland, 2017. https://www.research-collection.ethz.ch/entities/researchdata/bd1f72d2-1809-496e-8319-cf7c58f92a04.

[18] Carotenuto F., Bisignano A., Brilli L., Gualtieri G., Giovannini L. (2023). Low-Cost Air Quality Monitoring Networks for Long-Term Field Campaigns: A Review. Meteorol. Appl..

[19] Pineda-Tobón D.M., Espinosa-Bedoya A., Branch-Bedoya J.W. (2024). Aquality32: A Low-Cost, Open-Source Air Quality Monitoring Device Leveraging the ESP32 and Google Platform. HardwareX.

[20] Jafari S.N., Silva B.M.C. (2025). A Survey on Real-Time Data Transfer and Energy Consumption Strategies for Rural and Remote IoT Technologies. IEEE Open J. Comput. Soc..

[21] Reynders B., Meert W., Pollin S. (2017). Power and Spreading Factor Control in Low Power Wide Area Networks. Proceedings of the 2017 IEEE International Conference on Communications (ICC).

[22] Aljohani A., Rana O., Perera C. (2025). Self-Adaptive Federated Learning in Internet of Things Systems: A Review. ACM Comput. Surv..

[23] Alatawi M.N. (2025). SAFEL-IoT: Secure Adaptive Federated Learning with Explainability for Anomaly Detection in 6G-Enabled Smart Industry 5.0. Electronics.

[24] Ahmed R., Maddikunta P.K.R., Gadekallu T.R., Alshammari N.K., Hendaoui F.A. (2024). Efficient Differential Privacy Enabled Federated Learning Model for Detecting COVID-19 Disease Using Chest X-Ray Images. Front. Med..

